# Insights on hexavalent chromium(VI) remediation strategies in abiotic and biotic dual chamber microbial fuel cells: electrochemical, physical, and metagenomics characterizations

**DOI:** 10.1038/s41598-023-47450-9

**Published:** 2023-11-17

**Authors:** Dena Z. Khater, R. S. Amin, Amani E. Fetohi, Mohamed Mahmoud, K. M. El-Khatib

**Affiliations:** 1 Chemical Engineering and Pilot Plant Department, Engineering Research and Renewable Energy Institute, National Research Centre, 33 El-Buhouth St., Dokki, Cairo, 12311 Egypt; 2 Water Pollution Research Department, National Research Centre, 33 El-Buhouth St., Dokki, Cairo, 12311 Egypt; 3 Material and Manufacturing Engineering Department, Faculty of Engineering, Galala University, Galala City, Suez, 43511 Egypt

**Keywords:** Microbiology, Environmental sciences, Energy science and technology

## Abstract

Hexavalent chromium [Cr(VI)] is one of the most carcinogenic and mutagenic toxins, and is commonly released into the environemt from different industries, including leather tanning, pulp and paper manufacturing, and metal finishing. This study aimed to investigate the performance of dual chamber microbial fuel cells (DMFCs) equipped with a biocathode as alternative promising remediation approaches for the biological reduction of hexavalent chromium [Cr(VI)] with instantaneous power generation. A succession batch under preliminary diverse concentrations of Cr(VI) (from 5 to 60 mg L^−1^) was conducted to investigate the reduction mechanism of DMFCs. Compared to abiotic-cathode DMFC, biotic-cathode DMFC exhibited a much higher power density, Cr(VI) reduction, and coulombic efficiency over a wide range of Cr(VI) concentrations (i.e., 5–60 mg L^−1^). Furthermore, the X-ray photoelectron spectroscopy (XPS) revealed that the chemical functional groups on the surface of biotic cathode DMFC were mainly trivalent chromium (Cr(III)). Additionally, high throughput sequencing showed that the predominant anodic bacterial phyla were *Firmicutes*, *Proteobacteria,* and *Deinococcota* with the dominance of *Clostridiumsensu strict *1*, Enterobacter, Pseudomonas, Clostridiumsensu strict *11 and *Lysinibacillus* in the cathodic microbial community. Collectively, our results showed that the Cr(VI) removal occurred through two different mechanisms: biosorption and bioelectrochemical reduction. These findings confirmed that the DMFC could be used as a bioremediation approach for the removal of Cr(VI) commonly found in different industrial wastewater, such as tannery effluents. with simultaneous bioenergy production.

## Introduction

Hexavalent chromium [Cr(VI)] is considered one of the most carcinogenic and mutagenic toxins among numerous heavy metals and metalloids that are commonly found in various industrial waste streams, including electroplating, leather tanning, petroleum cleansing, pulp and paper manufacturing, and metal finishing^1–3^. The Commission of European Communities and the World Health Organization suggested that the maximum permissible limit for Cr(VI) is approximately 50 ppb^4^. Exposure to larger quantities of Cr(VI) causes acute toxicity effects of skin abscesses, lung, bladder, liver tumors, and other maladies in humans. Thus, an appropriate treatment step is usually required before discharging the effluents containing relatively high levels of Cr(VI) into the environment. Owing to the high toxicity and accumulation capability of Cr(IV) in the environment, various remediation technologies, including adsorption, electrocoagulation, photocatalytic reduction, and membrane separation, have been widely used to reduce toxic soluble Cr(VI) with rapid penetrability into less-toxic insoluble trivalent chromium [Cr(III)], which is considered an essential micronutrient for carbohydrate and fat metabolism in humans^5–7^.

Among several proposed treatment options, biological reduction has emerged as a sustainable, cost-effective remediation approach for removing toxic Cr(VI) compared to traditional remediation techniques owing to its simple operation, low energy consumption, easy management, high efficiency, and fewer secondary contamination problems^8^. In this context, microbial fuel cells (MFCs) have been recently proposed as an alternative remediation strategy for the biological reduction of Cr(VI) with simultaneous electricity production^9,10^. In a typical MFC, anodic electroactive biofilm is responsible for respiring the metabolically-produced electron from the anaerobic oxidation of organic matter, which is associated with the cathodic reduction of an electron acceptor (e.g., Cr(VI))^11,12^. For instance, Li et al.^13^ used a double chamber MFC equipped with an abiotic cathode for disposing of Cr(VI) in electroplating wastewater. Under acidic cathodic conditions (pH = 2), they observed up to 99.5% removal of Cr(VI) and 66.2% removal of total Cr with power density output being increased as a response to increasing Cr(VI) concentrations in the influent. More recently, Huang et al.^14^ revealed that a microbial electrochemical cell equipped with a biased biocathode (at − 0.3 V) outcompeted a control biocathode (with no applied potential) not only for achieving a high Cr(VI) reduction rate and higher power output, but also for shortening the start-up period (i.e., 19 days versus 26 days for control biocathode). Furthermore, Yeon et al.^15^ showed that enriching biofilm using inoculum contaminated with Cr(VI) was capable of achieving up to 93% removal of Cr(VI), which was associated with the emergence of phylotypes that are known for performing anode respiration and Cr(VI) reduction (i.e., *β-Proteobacteria*, *Actinobacteria*, and *Acinetobacter*). In another study, Xafenias et al.^16^ studied the poised biocathode enriched with *Shewanella oneidensis* MR-1 at − 0.5 V (vs. Ag/AgCl) was able to achieve up to 90% removal of Cr(VI) and produce a maximum current density of 32.5 mA/m^2^.

Our literature survey revealed that reactor architecture has a profound impact on the overall performance of MFCs, in terms of electric current generation, organic matter removal, and Cr(VI) reduction^17–19^. Although single-chamber MFCs seem to be a simple and low-cost configuration, they suffer from several limitations due to the absence of a membrane, including substrate consumption at the cathode and oxygen diffusion to the anode surface, resulting in low electron recovery and coulombic efficiency^20,21^. More seriously, the migration of toxic heavy metals, such as Cr(VI), toward the anode chamber could potentially cause serious inhibition of the anodic biofilm. In this context, dual-chamber MFC configuration has the potential to overcome these challenges, mainly due to the existence of an ion-selective separator^22^.

Since high Cr(VI) level could potentially modify microbial communities' structure and function, resulting in poor efficiency of MFCs, our study aimed to elucidate the role of biotic dual chamber MFC in comparison to abiotic dual chamber MFC for treatment of wastewater containing high concentrations of Cr(VI). Both biotic and abiotic MFCs were fed with different initial Cr(VI) concentrations, ranging from 5 to 60 mg/L, and their performance was evaluated, in terms of electricity generation, chemical oxygen demand removal, and of Cr(VI) reduction. In order to investigate the Cr(VI) bioreduction mechanism, we documented the change in the biofilm structure using high-throughput sequencing analysis. Finally, we also studied the change in the surface chemistry using X-ray photoelectron spectroscopy (XPS), scanning electron microscope (SEM), and energy dispersive spectroscopy (EDS) during the Cr(VI) bioreduction processes. Our results reveal that MFCs represent a promising technology for Cr(VI) reduction, and have the potential to be technically useful to regulate and optimize treatment processes of waste streams containing high concentrations of Cr(VI), such as tannery wastewater.

## Material and method

### MFCs configuration

Two dual-chamber MFC (DMFC) bioreactors were used to assess the removal efficiency of Cr(VI), which were assembled by connecting two plexiglass cylindrical chambers (6 cm long, 4.6 cm in diameter, and an effective volume of 100 mL) as cathodic and anodic chambers. We separated the anode and cathode chambers using a proton exchange membrane (PEM, Nafion 117, DuPont Co., USA). The anodes were carbon felt electrodes (Fuel Cell Store, TX, USA) with affective dimensions of 2.5 × 2.5 × 0.6 cm and a projected surface area of 18.50 cm^2^. The cathodes were made of a non-wet-proof gas diffusion carbon cloth with a microporous layer (6 cm × 6 cm and a projected surface area of 16.63 cm^2^). The anode chamber was completely sealed to keep an anaerobic environment, while the cathode chamber was left open to the atmosphere.

### MFCs operation

The anode chambers of biotic DMFCs were initially inoculated with anaerobic sludge from a local municipal wastewater treatment plant (Benha, Egypt), and operated in a fed-batch mode^23^. Following the bioreactor’s inoculation, the anode chamber was inoculated with growth medium containing (per liter): glucose: 1 g, NaHCO_3_: 2.5 g, NH_4_Cl: 0.2 g, H_2_PO_4_: 13.6 g, KCl: 0.33 g, NaCl: 0.3 g, K_2_HPO_4_: 17.4 g, CaCl_2_.2H_2_O: 0.15 g, MgCl_2_: 3.15, yeast extract 1 g, and 12.5 ml trace metal solution. The biocathodes were inoculated with the same inoculum similar to the anode chamber and growth medium except that glucose was replaced by NaHCO_3_ (0.2 g/L) as the sole inorganic carbon source. After the acclimation period, which lasted for 36 days, the catholyte was replaced with 50 mM of phosphate buffer electrolyte containing different Cr(VI) concentrations (i.e., 5–60 mg/L), mimicking tannery effluents^24,25^. In addition to the biotic DMFCs, two control reactors were also prepared. The first control reactor was a DMFC equipped with an abiotic cathode having Cr(VI) containing catholyte, while the other control reactor was a DMFC with an abiotic cathode having phosphate buffer catholyte. All DMFCs were operated in a fed-batch mode at room temperature (26 ± 2 °C), and we performed all experiments using two replicates.

### Electrochemical analyses

The MFC potential was montoried across an external resistor of 10 kΩ using a data acquisition system (U6 PRO, LabJack, Lakewood, CO, USA) connected to a personal computer. We estimated the current (mA m^−3^) and power densities (mW m^−3^) as described elsewhere^11,26^. Polarization plots were constructed by altering external resistors from 1 MΩ to 500 Ω, and the internal resistances (R_in_) were estimated from the slope of the linear region of polarization curves^27^. The coulombic efficiency (CE) was estimated by determining the ratio between the recovered electrons measured as current to the organic matter removed as follows^10^:

1 $${\varvec{CE}} = \frac{{{\varvec{M}}\mathop \int \limits_{0}^{{\varvec{t}}} {\varvec{Idt}}}}{{{\varvec{nFvC}}_{{{\varvec{Cr}}}} }} \times 100$$

where *I* is current output (A), *M* is the molecular weight of chromium (52 g mol^−1^), *v* is the working volume (L), *n* is the number of electrons exchanged per mole of Cr(VI) = 3, *F* is Faraday’s constant (96,485 A s mol^−1^), *C*_cr_ is the initial concentration of Cr(VI) (g L^−1^).

### Analytical and surface morphology analyses

We quantified Cr concentrations using inductively coupled plasma atomic emission spectroscopy (ICP-OES) (Agilent ICP-OES 5100, Australia) following an acid pre-treatment^28^, and its removal efficiency (Cr R%) was calculated according to Eq. (2):

2 $${\text{Cr R}}\% = \frac{{{\text{C}}_{{{\text{Cr}},{\text{ inf}}}} - {\text{ C}}_{{{\text{Cr}},{\text{ eff}}}} }}{{{\text{C}}_{{{\text{Cr}},{\text{ inf}}}} }} \times 100$$

where C_Cr,inf_ and C_Cr,eff_ are the Cr(IV) concentrations in MFC influent and effluent, respectively.

We documented the change in surface chemistry of cathode electrodes after exposing to high level of Cr using X-ray photoelectron spectroscopy (XPS) and field emission scanning electron microscopy (FE-SEM) equipped with energy-dispersive X-ray spectroscopy (EDX) mapping analyses.

### Microbial community analysis

Anodic and cathodic biofilm samples from biotic DMFC were entirely harvested using a pipette tip, and resuspended in a sterile centrifuge tube having DNA-free phosphate buffer. Biofilm samples were further concentrated using a centrifuge at 12,000*g* for 20 min. Then, we extracted genomic DNA using a DNA isolation kit (Qiagen, Germany) following the manufacturer's guidelines, and assessed its quality using a NanoDrop spectrophotometer (Thermo Scientific, USA). Finally, the MiSeq Illumina sequencer (Illumina Inc., USA) was used for high-throughput microbial community analysis, employing the bar-coded primer set according to the manufacturer's instructions as previously stated^21^.

Raw data was assigned by the amplicon analysis pipeline of the SILVA project (SILVAngs 1.4)^29^. Each read was aligned using the SILVA Incremental Aligner (SINA SINA v1.2.10 for ARB SVN (revision 21,008)). The reads shorter than 50 aligned nucleotides and reads with more than 2% of ambiguities, or 2% of homopolymers, respectively, were excluded from further processing. Putative contaminations and artifacts read with a low alignment quality (50 alignment identity, 40 alignment score reported by SINA), were identified and excluded from downstream analysis. After these initial steps of quality control, identical reads were identified, the unique reads were clustered (OTUs), on a per-sample basis, and the reference read of each OTU was classified. Dereplication and clustering were done using VSEARCH (version 2.17.0; https://github.com/torognes/vsearch)) for applying identity criteria of 1.00 and 0.7, respectively. Finally, the subsequent bioinformatics analyses were performed by BLASTn (2.11.0+; http://blast.ncbi.nlm.nih.gov/Blast.cgi) with standard settings using the non-redundant version of the SILVA SSU Ref dataset (release 138.1; http://www.arb-silva.de) as a classification reference. BLASTn had a sequence identity of more than 90% over an alignment of at least 50 bp. For each gene, the number of hits was normalized to the total number of sequencing reads, which yielded quantitative information (number of individual reads per taxonomic path)^30^. The sequence data sets can be accessed at NCBI/Sequence Read Archive (SRA) under the study designated by the BioProject accession number: PRJNA1011343.

## Results and discussions

### Adaptation of the DMFCs

**Figure 1:**
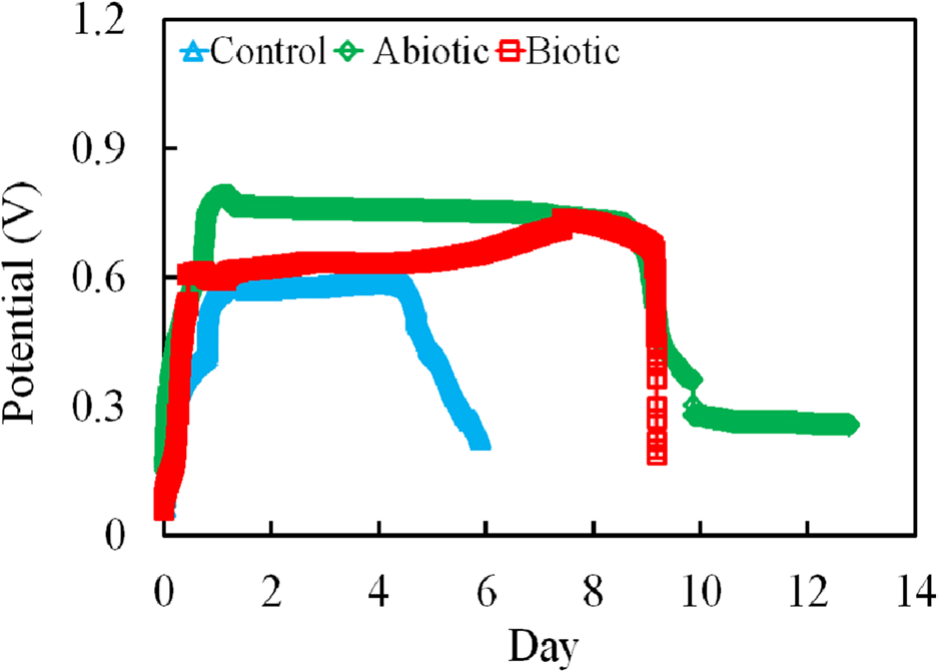
Open circuit potential of MFC bioreactors.

Following the successful acclimation period for the formation of anodic and cathodic biofilm communities, the DMFCs were fed with glucose medium (1 g/L) and operated in open circuit potential (OCP) condition as shown in Fig. 1. It can be noted that the voltage development was gradually increased during the stable operation phase. The overall OCP production after three cycles in biotic cathode DMFC, abiotic cathode DMFC, and control (without addition) were 0.706 ± 0.107, 0.662 ± 0.106, and 0.436 ± 0.161 V, respectively. Generally, OCP shows the maximum theoretical potential that can be generated during a preferred operating condition, which is commonly used to evaluate the possibility of MFCs for generating high power output from organic substrate oxidation^31,32^. Our results revealed that biotic cathode DMFC exhibited a higher possibility for generating electricity compared to other tested bioreactors owing to higher stability, and minimal energy losses and overpotential in both anodic and cathodic electrodes^33^.

### Performance under an incremental increase in Cr(VI) concentrations

**Figure 2:**
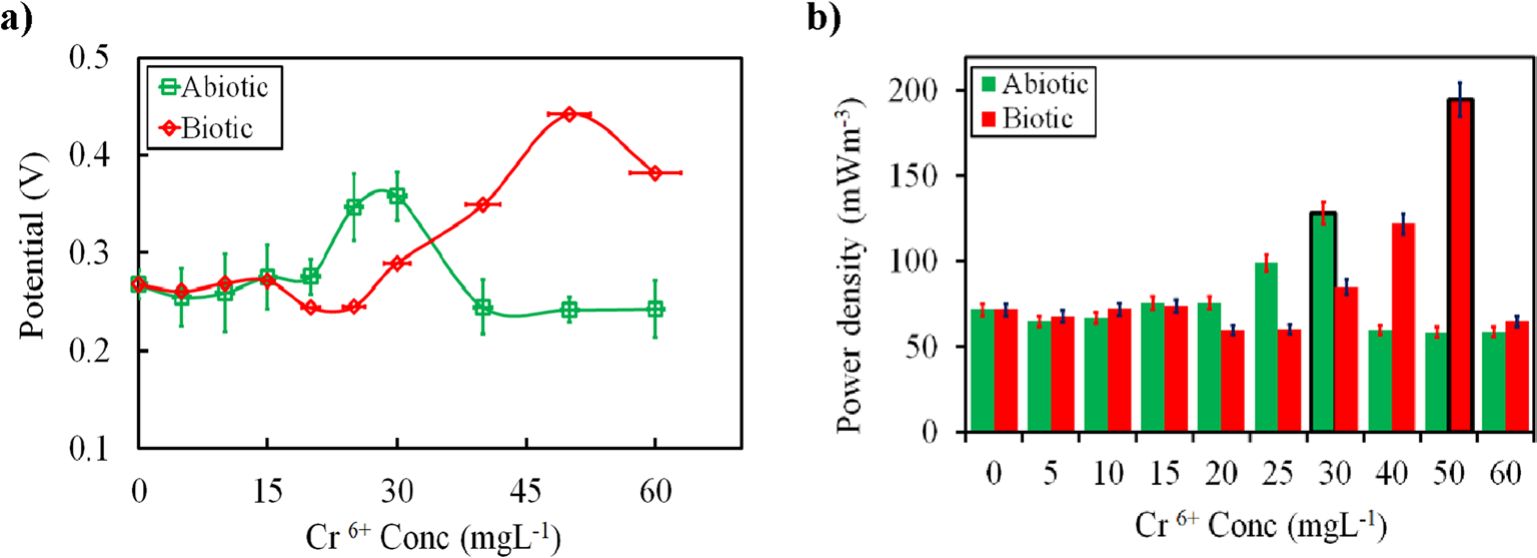
The effect of initial Cr^6+^ concentration (from 5 to 60 mg L^−1^) on the (**a**) closed-ciruit potential generation and (**b**) power density output. Error bars indicate the relative standard deviation of two replicates.

The maximum tolerable concentrations (MTCs) and the maximum Cr(VI) concentrations that do not cause inhibition for MFCs were estimated in order to investigate the effects of the Cr(VI) concentration on electricity generation. In this context, MFCs were fed with growth media having different initial concentrations of Cr(VI) in the range of 5–60 mg L^−1^ at a fixed external resistance of 10 kΩ. As observed in Fig. 2, the MTCs for abiotic and biotic cathode DMFCs were estimated as 30 and 50 mg L^−1^, respectively. Moreover, it could be observed that the maximum voltage (0.36 ± 0.01 V) corresponding to a maximum volumetric power density (PD_max_) of 128.17 ± 35 mW m^−3^ at an initial Cr(VI) concentration of 30 mg L^−1^, which was dropped by approximately 1.5-fold when the abiotic cathode DMFC was fed with the medium solution containing 60 mg Cr(VI) L^−1^. However, in the case of biotic cathode DMFC, the PD_max_ was much higher (i.e., 195.1 ± 0.41 mW m^−3^) with a maximum voltage of 0.44 ± 0.01 V at an initial Cr(VI) concentration of 50 mg L^−1^, indicating more functional stability for biotic cathode DMFC compared to abiotic cathode DMFC. Similar to abiotic cathode DMFC, further increase in the influent Cr(VI) concentration resulted in a decrease in the overall MFC performance, implying that high influent Cr(VI) concentrations caused partial inhibition for electroactive biofilm. Therefore, the minimum inhibitory concentrations (MICs) were 40 and 60 for abiotic and biotic cathode DMFC, respectively. It could be concluded that, in the case of biotic cathode DMFC, the microbes were adapted to medium solutions containing Cr(VI) and became more resistant to the toxicity of Cr(VI) at lower concentrations. Nevertheless, at concentrations higher than MTCs, resulted in a much slower decrease in voltage output, according to the acute toxicity of Cr(VI) that inhibits the growth of microorganisms and irreversible damage to microbial DNA^34–36^. While, in the case of abiotic cathode DMFC, the output was limited after its MTC due to the electrostatic repulsion between the carbon cloth and Cr(VI) that blocked the Cr(VI) anions from moving near the cathode.

### Determination of coulombic efficiency and Cr(VI) removal efficiencies

**Figure 3:**
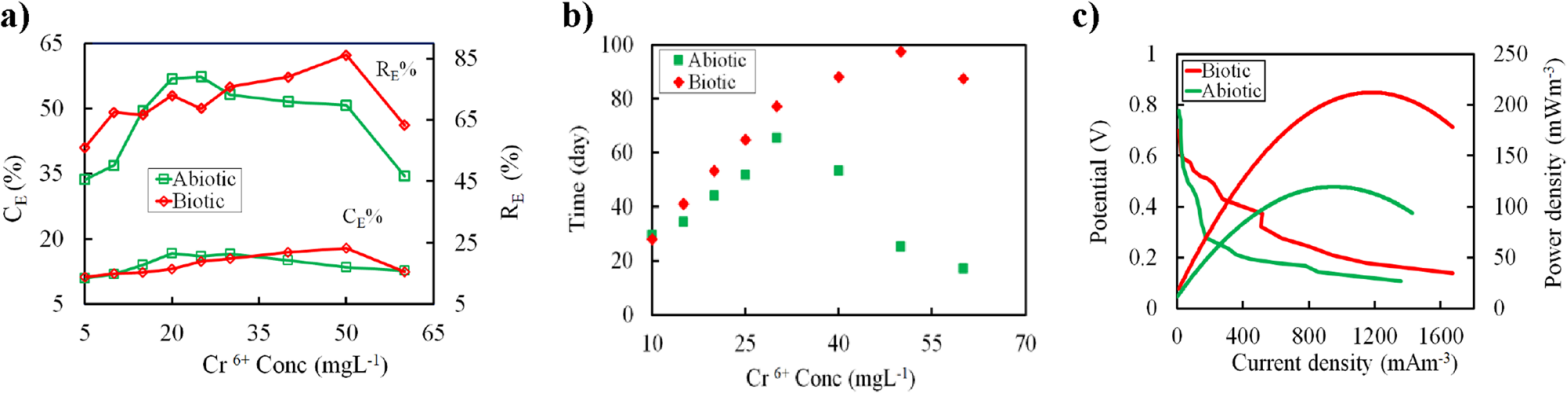
(**a**) Coulombic efficiency (C_E_) and removal efficiencies (R_E_), (**b**) adaptation of microbial community over time and different Cr(VI) concentrations, and (**c**) power and polarization curves for abiotic and biotic cathode DMFC reactors during the experimental period at 10 KΩ resistance.

Figure 3a showed that coulombic efficiency (C_E_) was peaked for both biotic cathode DMFC (i.e., 17.88%) and abiotic cathode DMFC (i.e., 16.57%) MFCs at Cr(VI) concentration lower than MTCs, and there were significant drops at concentrations over the MTCs. Consistent with C_E_ results, we observed that biotic cathode DMFC exhibited significantly higher Cr(VI) removal efficiency (i.e., 86.25%) compared to abiotic cathode DMFC (73.3%) at an initial Cr(VI) concentration of 30 and 50 mg L^−1^, respectively. These results documented that a higher Cr(VI) bioreduction rate was detected in biotic DMFCs, likely due to the formation of mature biocathode communities that acted as bioelectrocatalysts^37^.

The influence of different Cr(VI) concentrations on the microbial communities’ biomass generation and adaptation over time was illustrated in Fig. 3b. It could be observed that the bacterial communities sustained to develop with an increase in Cr(VI) concentration. Moreover, the removal of Cr(VI) was accomplished successfully within 98 and 65 days at maximum Cr(VI) reduction of 50 and 30 mg L^−1^ for biotic and abiotic cathode DMFC, respectively, which was considered as the final Cr(VI) concentration for the viability of microbial communities. The removal of Cr(VI) sustained from an initial concentration of 5–50 mg L^−1^ and the overall removal rate was decreased gradually. Thus, the bacterial communities were able to grow at this range of concentrations. Additionally, the increase in Cr(VI) up to 50 mg L^−1^ for biotic cathode DMFC and 30 mg L^−1^ for abiotic cathode DMFC resulted in a sudden inactivity and inhibition in substrate consumption and electricity generation over time, which directly affected Cr(VI) removal. Thus, our results revealed that the overall MFC's performance was primarily correlated to the toxicity of Cr(VI) towards cathodic microbes rather than competing for electron acceptors in MFCs, which are in agreement with previous studies^38–42^.

### Polarization and power outputs

The performance of DMFCs was evaluated by systematically adjusting the external resistance within the range of 1 MΩ to 500 Ω to develop polarization and power curves, which provide insights into the behavior of the DMFCs (Fig. 3c). It could be observed that the maximum volumetric power density of the biotic cathode DMFC was 195.60 mW m^−3^, which was 1.5 times higher than that of the abiotic cathode DMFC (128.93 mW m^−3^) at a current density of 1671.43 and 780.95 mA m^−3^, respectively. The obtained power density in our study was slightly lower than previous studies, which showed a maximum power density in the range of 55.5–1965.4 mW m^−2^ (Table 1). The difference in performance can be attributed to several factors, such as different electron donors, DMFC configuration, electrode materials, inoculum type, Cr(VI) concentration, and separator types. The internal resistance (R_in_) of the biotic cathode DMFC was 598 Ω, which was significantly lower than that of the abiotic cathode DMFC (i.e., 900 Ω). Our results revealed that the biocathode had a profound impact on the overall performance of MFCs enriching resilient microbial communities that have the capability to reduce Cr(VI) and reduce the R_in_ for improving electricity generation in MFCs. The lower efficiency of the abiotic cathode DMFC could be due to the electrostatic repulsion between the carbon cloth and Cr_2_O_7_^−2^ ions, resulting in poor Cr(VI) conversion efficiency and coulombic efficiency^43^.

**Table 1:** Summary of bioelectrochemical removal of Cr(VI) in dual chamber MFC relevant to the present work.

Electron donor	Cr(VI) Concentrations (mg L^−1^)	Removal efficiency	Power density	References
Acetete	5–60	86.25%	16.94 mWm −^2^	Current study
Acetate	22–63	0.30 mg Cr(VI)/g VSS h	360.0 mWm −^3^	^44^
Formate	40–100	70.7%	1965.4 mWm −^2^	^10^
Industrial waste	5 and 10	–	75.08 mWm −^2^	^24^
Glucose	6–20	59.1%	55.5 mWm −^2^	^45^
Lactate	20	35%	299.6 mWm −^2^	^46^

### Morphological and molecular characterization of the cathodes

#### XPS analysis

**Figure 4:**
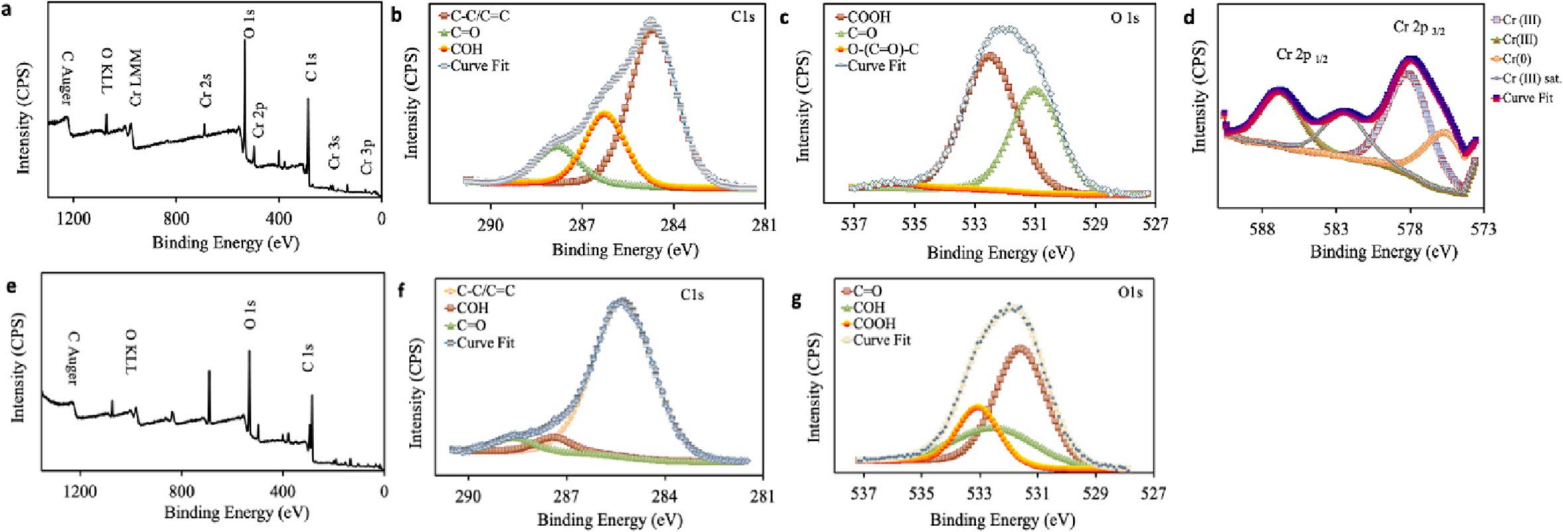
XPS spectra of (**a**) XPS survey spectrum of biotic cathode and high-resolution XPS spectra of (**b**) C 1s, (**c**) O 1s, and (**d**) Cr2p. (**e**) XPS survey spectrum of abiotic cathode and high-resolution XPS spectra of (**f**) C 1s and (**g**) O 1s.

In order to understand the main mechanism for the bioreduction of Cr(VI), we used XPS analysis to evaluate the change of surface properties and chemical functional groups of cathode electrodes for both abiotic and biotic cathode DMFC. From the wide XPS spectra survey (as shown in Figs. 4a,e), it could be noticed that there were four major peaks at binding energies of 286.82, 533.33, 401.25, and 192.16 eV, which were correlated to C 1s, O 1s, N 1s, and Cl 2p, respectively^47^. Furthermore, the presence of a weak peak of Cr 2p only for biotic cathode DMFC at binding energies of 591.46 eV demonstrated that the chromium was deposited on the cathode surface. In addition, it could be observed that the C 1s spectra (Fig. 4b,f) were fitted to the main three characteristic peaks at binding energies of 284.95 (C–C/C=C), 286.57 (C–OH) 288.35 (C=O) eV^48^. Moreover, in the case of O1s spectra, as illustrated in (Fig. 4c,g), the absorption peaks were deconvoluted into three single peaks with binding energies of 531.25, 532.34, and 533.79 eV corresponding to C=O, O–H, and O–C=O, respectively^49^.

The Cr2p XPS spectra region of biotic cathode DMFC as shown in Fig. 4d illustrated that the binding energies of Cr 2p_1/2_ and Cr 2p_3/2_ showed two obvious peaks at 582.38 and 574.6 eV, respectively. These two distinct peaks were noticed at binding energies of 577.66 eV (Cr 2p_3/2_) and 586.77 eV (Cr 2p_1/2_) that were assigned to the presence of trivalent (Cr^3+^) with an atomic ratio of 79.94%. Furthermore, another peak at around 574.91 eV corresponded to the elemental Cr^0^, while the peak at 582.38 eV was assigned to Cr (sat). It could be concluded that the bioabsorption of Cr^3+^compounds likely occurred with the C–H, C–C, C=O, C–OH, and O–C=O of bacterial cells for biotic cathode DMFC. Also, the binding of Cr^3+^ to the cell surface principally existed as Cr(OH)_3_ and Cr_2_O_3_ forms^8,50^.

#### Functional analysis of differential genes associated with cytotoxicity of Cr(VI)

**Figure 5:**
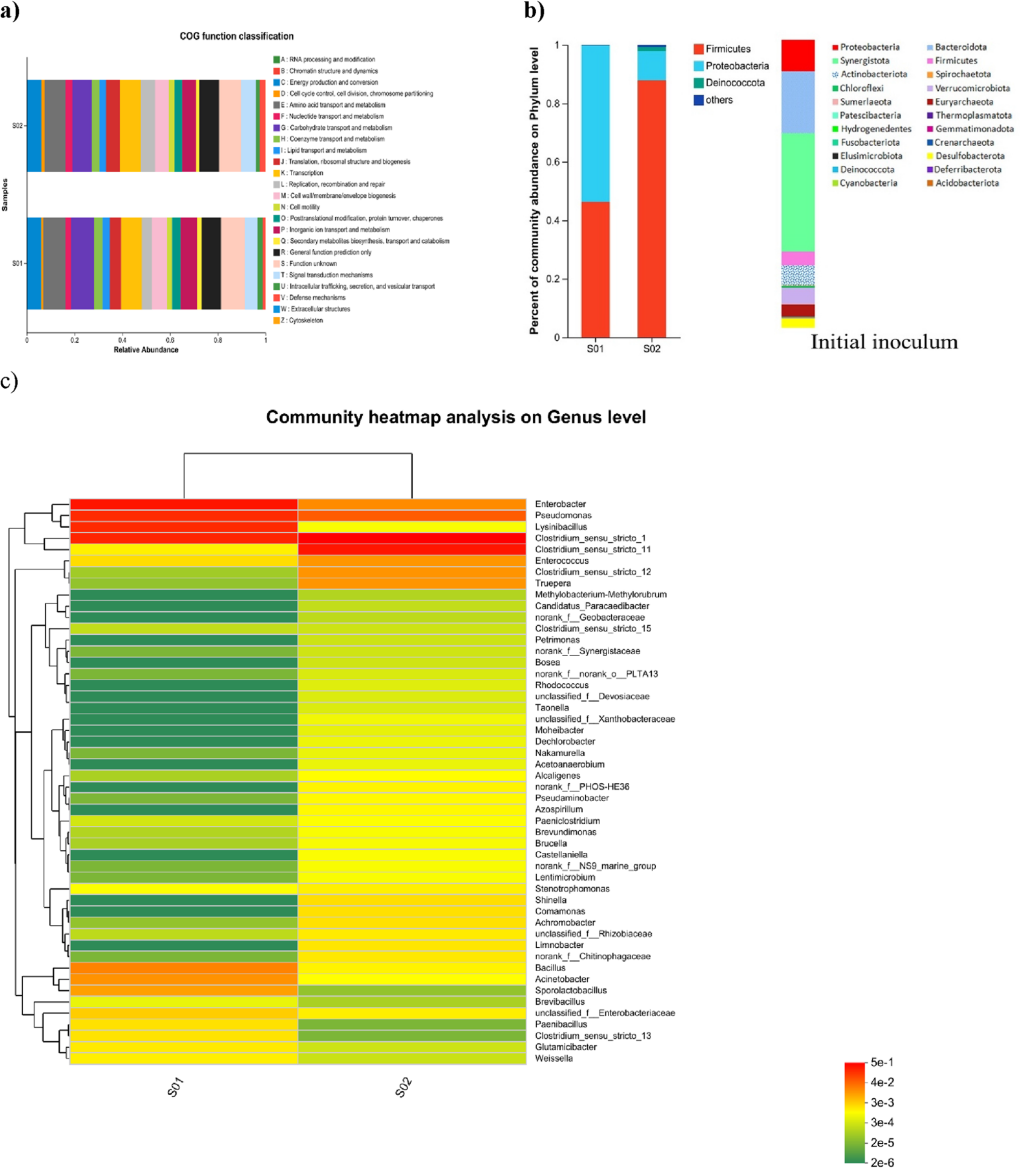

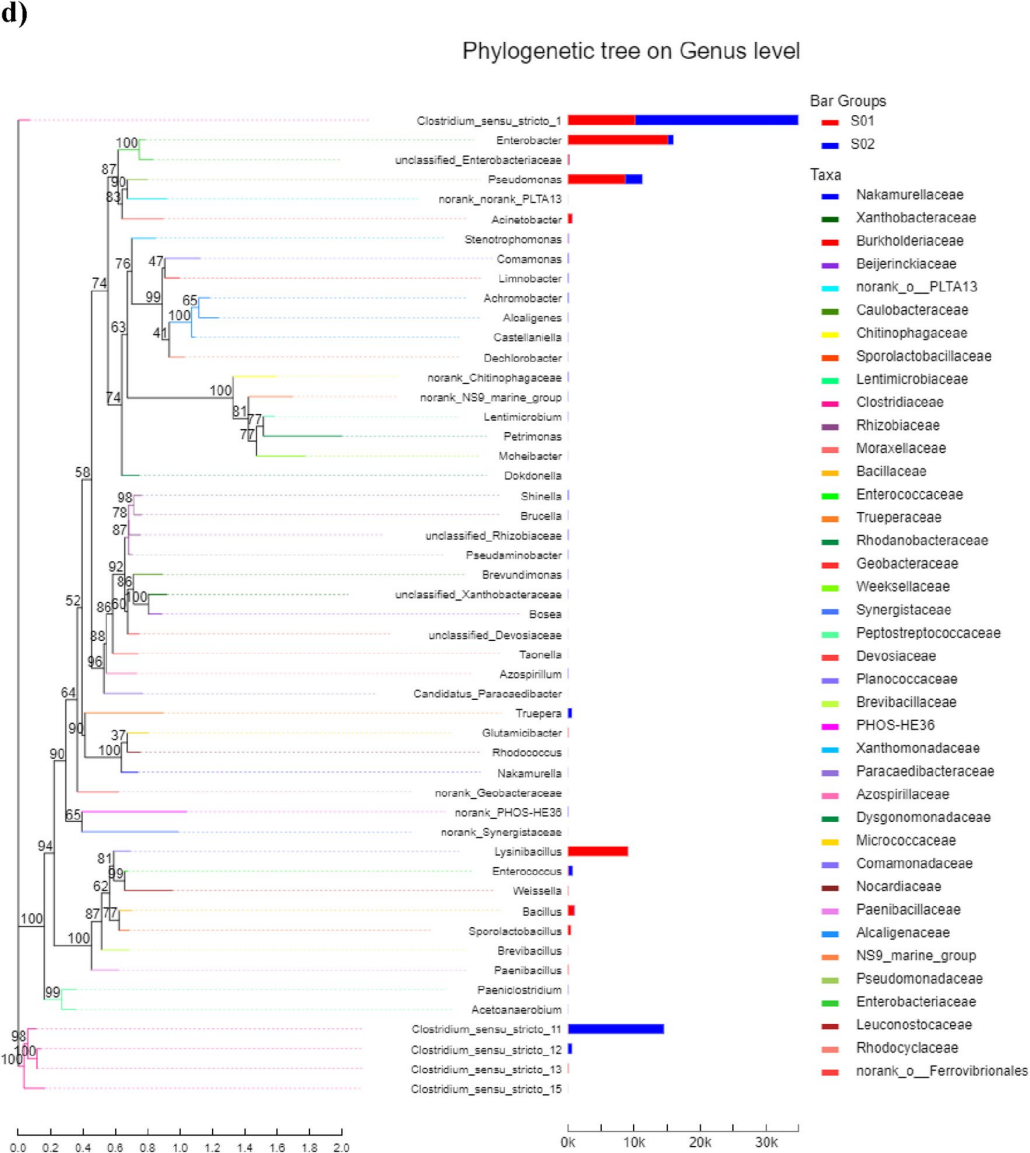
Microbial diversity analysis related to Cr(VI) cytotoxicity: (**a**) the functional analysis of expressed genes using PICRust, (**b**) relative abundance of bacterial community sequencing results at the phylum level (Phylotypes < 1% of total sequences were classified as “others”), (**c**) Heat map depicting the comparison of the relative abundance of the dominant microbial genera, and (**d**) Phylogenetic tree at genus level of biocathodic and anodic biofilms in biotic MFC.

The comprehensive functional analysis of differential genes for the biocathode surface of biotic DMFC (S01) at its MTCs and its anodic biofilm (S02) was conducted to gain deeper insight into the main functions and expressed genes associated with the cytotoxicity of Cr(VI). The significant biological process for both communities is shown in Fig. 5a. The considerable COG analysis of differential genes showed that the most prominent biological process and molecular analysis were chromatin structure and dynamics, energy production and conversion, cell cycle control, cell division, chromosome partitioning, amino acid transport and metabolism, nucleotide transport and metabolism, carbohydrate transport and metabolism, coenzyme transport and metabolism, lipid transport and metabolism, translation, ribosomal structure and biogenesis, transcription, replication, recombination and repair, cell wall/membrane/envelope biogenesis, cell motility, post-translational modification, protein turnover, chaperones, inorganic ion transport and metabolism, secondary metabolites biosynthesis, transport and catabolism, general function prediction only, function unknown, signal transduction mechanisms, intracellular trafficking, secretion, and vesicular transport, defense mechanisms, extracellular structures, cytoskeleton.

#### Bacterial community analysis

The microbial diversity profiling was carried out to determine whether a differentiating impact could be observed due to the presence of Cr(VI) in biocathode biofilm (S01) and its percentages were compared to anodic biofilm (S02) in biotic DMFC. Figure 5b shows the microbial diversity based on the phylum levels of both the biofilm compared to the inoculum. It could be observed that significant changes in the number of phyla diversity in the initial inoculum and the formed biofilms. Furthermore, the desertion of more phyla in the initially inoculated sludge after Cr(VI) addition. The predominant phyla for both biofilms were *Firmicutes*, *Proteobacteria*, *Deinococcota*, *Bacteroidota*, and *Actinobacteriota* but with different proportions in response to the Cr(VI) raised concentration. The majority of sequences (anodic biofilm (S02): 85% and biocathodic biofilm (S01): 48%) belonged to *Firmicutes* whereas the predominant *Proteobacteria* occupied 50% and 10% in the anodic and cathodic biofilms, respectively. In addition, the anode is more preferred for *Deinococcota* (3%), *Bacteroidota* (1%), and *Actinobacteria* (1%) than the 1%, 0.5%, and 0.5% with the cathode, representing susceptibility of these phyla by various MFCs condition.

At the Genus level, as displayed in the heatmap, high-throughput sequencing of 16S rRNA gene suggested the occurrence of 49 genera of the bacterial community in both biofilms, but in different proportions (Fig. 5c). *Clostridium *sensu* strict 1* (50%) and *Clostridium *sensu* strict 11* (32%) were predominantly present on the anodic biofilm, compared to the dominance of *Enterobacter (35*%*)*, *Lysinibacillus* (20%) and *Pseudomonas (15*%) in the cathodic biofilm. Other abundant genera including *Clostridium *sensu* strict 12(3*%*), Enterococcus* (3%), and *Truepera*(2%) were observed in anodic biofilm, while *Acinetobacter(6*%*), Bacillus* (5%), and *Sporolactobacillus* (1%) were present in the cathodic biofilm, besides the presence of anonymous genera from environmental samples. To further explore the dominant genera involved in the removal process, the phylogenetic lineage exploration was constructed from the 16s rRNA sequence. As seen in Fig. 5d, the amplified genomic cluster even displayed similarity closely related to *Clostridiumsensu strict 1* (100%)*, Enterobacter* (100%)*, Pseudomonas* (90%)*, Clostridiumsensu strict 11*(98%)*,* and *Lysinibacillus* (81%). The Firmicutes and Proteobacteria phyla and their species such as *Pseudomonas, Enterobacter, Bacillus,* and *Clostridium* were also recognized in other previous literature for the bioreduction of Cr(VI) into Cr(III)^37,44,51^. Our high-throughput sequencing demonstrates the efficient formation of a highly diverse biocathode that has the capability to efficiently reduce Cr(VI). In addition, anodic electroactive microbial communities played a vital role in the oxidation of glucose, and respire the resulting electrons into the anode surface without the need for external mediators. Consequently, internal resistance was significantly reduced, resulting in achieving high power density and Cr(VI) reduction^52–54^.

#### FE-SEM–EDX analyses of the cathodes after MICs

**Figure 6:**
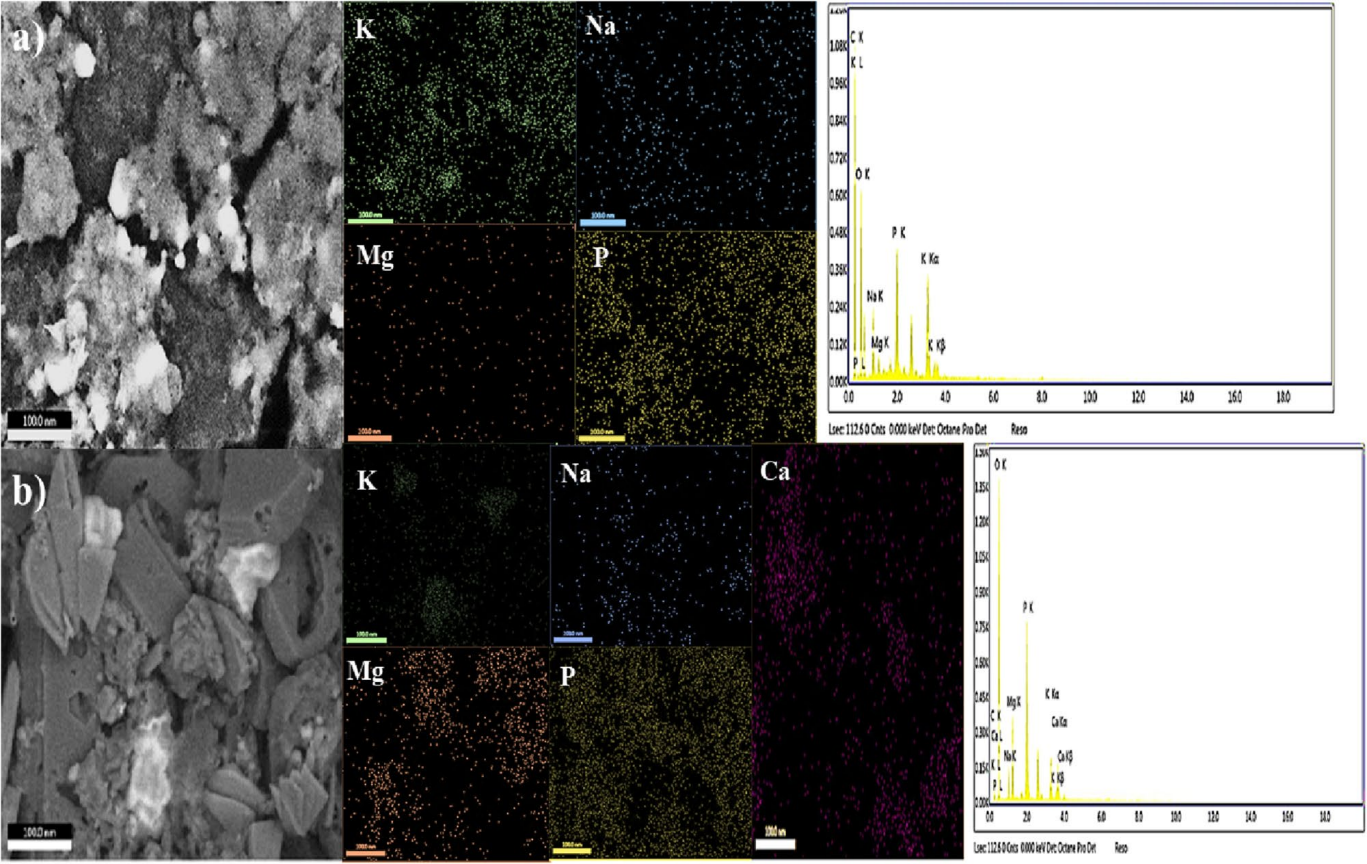
SEM analysis and EDX spectra coupled with elemental mapping of (**a**) abiotic and (**b**) biotic cathode DMFCs.

FE-SEM coupled with EDX mapping analyses of abiotic and biotic cathode electrodes were performed to explore the morphological changes of cathode electrodes after 6 months of operation (Fig. 6). It could be observed that there was an accumulation of amorphous non-biodegradable cations according to the electrostatic interactions that facilitate metal cations to transfer to the cathode (carbon cloth) that had a negative effect on the performance of abiotic cathode DMFC towards the Cr(VI) removal. Furthermore, we observed the absence of microbial colonization or broken biofilm on the cathode of biotic cathode DMFC. EDX was further performed to confirm the chemical elements’ compositions for the aggregated amorphous particles on the electrode's surface as indicated in Table 2. We observed that the characteristic peak of the elemental Cr was not detected, which could be due to its solubility in buffer solution and the MFCs became unable to reduce Cr(VI) into Cr(III) according to the accretion of excess non-biodegradable chemicals and other inorganic chemicals in the surface of cathodic electrodes. Other elements such as C, O, Na, Mg, Ca, P, and K were also detected by elemental mapping with high contents. These elements usually affected the long-term performance of MFCs and confirmed that the Cr(VI) reduction was related to the present bacterial communities on the surface of cathode electrodes for biotic cathode DMFC through biosorption, not the precipitation process.

**Table 2:** Weight and atomic variation percentages of the elemental composition of MFCs electrodes.

Element	Abiotic MFC		Biotic MFC	
	Weight %	Atomic %	Weight %	Atomic %
C K	48.27	59.06	9.77	14.8
O K	36.37	33.42	57.28	65.13
MgK	0.54	0.33	6.25	4.67
P K	4.77	2.26	13.16	7.73
K K	5.68	2.13	4.35	2.02
CaK	–	–	4.85	2.2
NaK	4.37	2.8	4.34	3.44

### Proposed mechanisms

**Figure 7:**
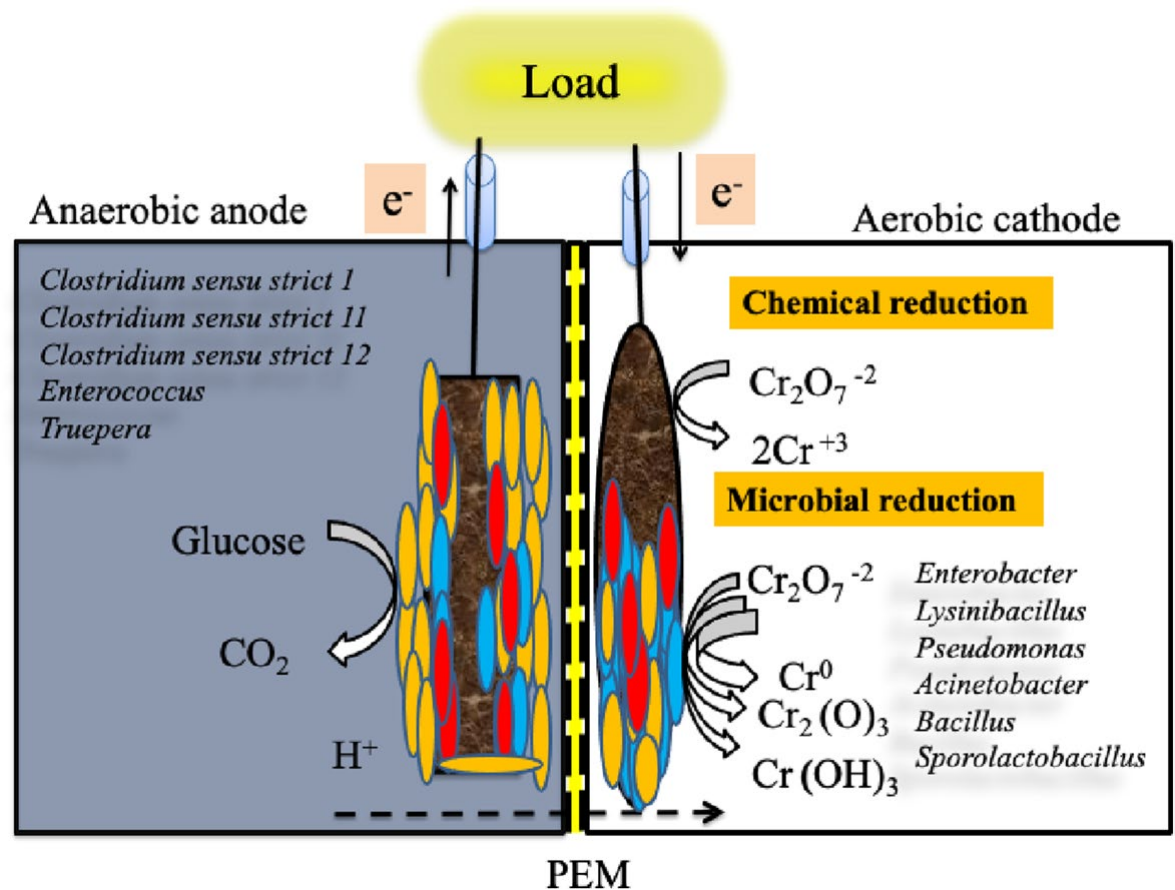
Proposed mechanism for Cr(VI) reduction in MFCs.

Our results showed that Cr(VI) reduction in MFCs proceeds through various routes. As illustrated in Fig. 7, the first mechanism involved the direct chemical reduction of Cr(VI) on the abiotic cathode into Cr(III) that might be directly used as electron acceptors without the need for an external power supply as follows^55,56^:

3 $${\text{Cr2O}}_{7}^{ - 2} + 14{\text{H}}^{ + } + 6{\text{e}}^{ - } \to 2{\text{Cr}}^{ + 3} + 7{\text{H}}_{2} {\text{O}}\,\,\,\,\,{\text{E}}^{ \circ } = + 1.33\,\,{\text{V}}\,{\text{(SHE)}}$$

The above half-cell reaction (E^o^ = + 1.33 V) specified the superior affinity of reduced Cr(VI) that could be used as a cathodic electron acceptor in the MFCs for power generation and the treatment of Cr(VI)-containing wastewater^9^. The second mechanism occurred by the bioelectrochemical reduction of toxic Cr(VI) with the assistance of microbial metabolisms in biotic cathodes, which involved the biosorption of non-toxic and less mobile form of Cr (i.e., Cr(III) and/or Cr^0^) on the cathode surface. Although the abiotic cathode seems to be more promising for MFCs scaling up compared to biocathode-based MFCs owing to its easy handling and low probability for performance decline due to exposure to high Cr(VI) levels, our results show great promise of biocathode-based MFCs not only to reduce Cr(VI) up to 50 mg L^−1^, but also to produce high power density, making MFCs a promising bioremediation technology for removing Cr(VI) from contaminated water bodies.

### Outlook and future considerations

Although MFCs represent a promising biotechnology for generating renewable energy from waste streams, their relatively high cost and low substrate-to-electricity conversion rate limit their commercialization and scaling up, particularly for toxic metals-containing waste streams^57,58^. Thus, the commercialization of MFCs should aim to enhance the substrate conversion into electricity and to find relatively cheap materials for the reactor’s construction without compromising the performance for large-scale and long-term operation. In this study, we used relatively cheap carbon-based electrodes, which have high flexibility, surface area, electrical conductivity, and mechanical stability in comparison to metal-based electrodes, making them suitable electrode materials for both anodic and cathodic chambers. Furthermore, the hallmark of our system is the use of biocathode without any precious metal cathodic catalysts, which commonly accounts for ~ 50% of the overall cost of MFCs^59–61^. Given the ongoing research in material science and engineering, we expect that the overall cost of materials used in MFCs could be potentially reduced. Furthermore, energy demand for powering pumps, motors, and other electric-based operations, which usually account for a large portion of the wastewater treatment operating cost, can be substantially decreased by the in-situ electricity generation from MFCs. Therefore, our developed biotic DMFC system opens up opportunities for simultaneous recovery of Cr(VI) and electricity generation from different industrial wastes, such as tannery wastewater and electroplating industrial wastewater, which is considered a challenge for future developments of MFCs.

## Conclusion

Cr(VI) represents one of the most carcinogenic and mutagenic toxins among numerous heavy metals and metalloids that are commonly found in various industrial waste streams, including electroplating, leather tanning, petroleum cleansing, pulp manufacture, and metal finishing. This study aimed to develop a highly efficient bioremediation approach with simultaneous electricity generation using MFCs. Our results showed that biotic-cathode DMFC achieved significantly higher power density (195.1 ± 0.41 mW m^−3^) and Cr(VI) reduction (86.25%) compared to abiotic-cathode MFC (128.1 ± 0.35 mW m^−3^ and 73.3%, respectively). More importantly, the MTCs and MICs were found to be 30 mg L^−1^ and 40 mg L^−1^, and 50 mg L^−1^ and 60 mg L^−1^ for abiotic-cathode DMFC and biotic-cathode DMFC, respectively, implying the beneficial role of biocathodes in alleviating the toxicity due to high Cr(VI) concentrations. Additionally, XPS investigation revealed that the chemical functional groups on the surface of biotic cathode DMFC were mainly trivalent chromium (Cr(III)). High throughput sequencing showed the formation of robust, highly diverse anodic and cathodic biofilm, resulting in higher power output. Furthermore, our results demonstrated that the Cr(VI) removal proceeded through 2 main mechanisms: biosorption and bioelectrochemical reduction. Collectively, this study provides a new insight into Cr(VI) reduction in MFCs, which would extend our knowledge for the future techno-economically feasible application of using MFCs for the treatment of industrial wastewater containing heavy metals, such as Cr.

## Data Availability

Source data that support the findings of this study were presented and provided in the main article. The raw data used in this study were uploaded to the NCBI/Sequence Read Archive (SRA) under Bioproject Accession Number: PRJNA1011343.
